# Aggrecan governs intervertebral discs development by providing critical mechanical cues of the extracellular matrix

**DOI:** 10.3389/fbioe.2023.1128587

**Published:** 2023-03-02

**Authors:** Marta Empere, Xujia Wang, Carina Prein, Anders Aspberg, Markus Moser, Toshitaka Oohashi, Hauke Clausen-Schaumann, Attila Aszodi, Paolo Alberton

**Affiliations:** ^1^ Musculoskeletal University Center Munich (MUM), Department of Orthopaedics and Trauma Surgery, Ludwig-Maximilians-University (LMU), Munich, Germany; ^2^ Center for Applied Tissue Engineering and Regenerative Medicine, Munich University of Applied Sciences, Munich, Germany; ^3^ Rheumatology and Molecular Skeletal Biology, Department of Clinical Sciences Lund, Lund University, Lund, Sweden; ^4^ Department of Molecular Medicine, Max Planck Institute of Biochemistry, Max Planck Society, Martinsried, Germany; ^5^ Institute of Experimental Hematology, School of Medicine, Technische Universität München, Munich, Germany; ^6^ Department of Molecular Biology and Biochemistry, Okayama University Graduate School of Medicine, Dentistry and Pharmaceutical Sciences, Okayama, Japan

**Keywords:** aggrecan, intervertebral disc, development, biomechanical properties, extracellular matrix, atomic force microscopy

## Abstract

Aggrecan (ACAN) is localized in the intervertebral disc (IVD) in unique compartment-specific patterns where it contributes to the tissue structure and mechanical function together with collagens. The extracellular matrix (ECM) of the IVD undergoes degenerative changes during aging, misuse or trauma, which inevitably alter the biochemical and biomechanical properties of the tissue. A deeper understanding of these processes can be achieved in genetically engineered mouse models, taking into account the multifaceted aspects of IVD development. In this study, we generated aggrecan insertion mutant mice (*Acan* ^*iE5/iE5*^) by interrupting exon 5 coding for the G1 domain of ACAN, and analyzed the morphological and mechanical properties of the different IVD compartments during embryonic development. Western blotting using an antibody against the total core protein failed to detect ACAN in cartilage extracts, whereas immunohistochemistry by a G1-specific antibody showed weak signals in vertebral tissues of *Acan* ^*iE5/iE5*^ mice. Homozygous mutant mice are perinatally lethal and characterized by short snout, cleft palate and disproportionate dwarfism. Whole-mount skeletal staining and µ-CT analysis of *Acan* ^*iE5/iE5*^ mice at embryonic day 18.5 revealed compressed vertebral bodies with accelerated mineralization compared to wild type controls. In *Acan* ^*iE5/iE5*^ mice, histochemical staining revealed collapsed extracellular matrix with negligible sulfated glycosaminoglycan content accompanied by a high cellular density. Collagen type II deposition was not impaired in the IVD of *Acan* ^*iE5/iE5*^ mice, as shown by immunohistochemistry. Mutant mice developed a severe IVD phenotype with deformed nucleus pulposus and thinned cartilaginous endplates accompanied by a disrupted growth plate structure in the vertebral body. Atomic force microscopy (AFM) imaging demonstrated a denser collagen network with thinner fibrils in the mutant IVD zones compared to wild type. Nanoscale AFM indentation revealed bimodal stiffness distribution attributable to the softer proteoglycan moiety and harder collagenous fibrils of the wild type IVD ECM. In *Acan* ^*iE5/iE5*^ mice, loss of aggrecan resulted in a marked shift of the Young’s modulus to higher values in all IVD zones. In conclusion, we demonstrated that aggrecan is pivotal for the determination and maintenance of the proper stiffness of IVD and vertebral tissues, which in turn could play an essential role in providing developmental biomechanical cues.

## 1 Introduction

The intervertebral disc (IVD) is a composite structure that provides structural stability to the vertebral column while allowing its rotation, lateral bending and axial compression (Hickey and Hukins, 1980). The mature IVD consists of three anatomically distinct parts: 1) the central, gelatin-like nucleus pulposus (NP), 2) the peripheral, fibrocartilaginous annulus fibrosus (AF) in the dorsal-ventral direction, and 3) the hyaline-like cartilaginous end plates (CEPs) rostrally and caudally, which are continuous with the adjacent vertebral bodies (VB) (Eyre, 1979; Buckwalter, 1995). The NP is designed to resist compressive forces due to its high proteoglycan content (Hutton et al., 2000), while the peripheral lamellar AF is rich in collagen and thus provides tensile strength to the IVD. The thin CEPs structurally and functionally resemble to the articular cartilage of the synovial joints and regulates nutrient diffusion and load distribution between the disc and the vertebrae (Urban et al., 2004; MacLean et al., 2007). The distinct composition, structure and biomechanical properties of the three IVD compartments enable the spinal column with flexibility and efficient dissipation of the mechanical load. The material and functional properties of the IVD compartments are largely conserved across species, however, their histological appearance may differ between animals and humans (O’Connell et al., 2007; Beckstein et al., 2008; Zhang et al., 2014).

The IVD experiences remarkable structural and compositional changes during development (Antoniou et al., 1996; Melrose et al., 2001). Genetic and grafting experiments have demonstrated the central, signaling role of the notochord, a transient, rod-like axial structure in the formation of the vertebral column. Early in development, the notochord triggers the differentiation of the sclerotome in the ventral part of the paraxial somites and induces the migration of sclerotomal cells towards the notochord, where they acquire a metameric condensation pattern (Smith et al., 2011; Shah et al., 2017; Tani et al., 2020). In mouse at embryonic day 12.5 (E12.5), the less condensed pre-vertebral (PV) region and the more condensed intervertebral mesenchyme (IM) later differentiate into the vertebral bodies and the AF of the IVD, respectively. AF cells are elongated and form a lamellar structure around the notochord with distinct inner and outer layers. The inner AF is cartilaginous with chondrocyte-like cells depositing type II collagen and proteoglycans, whereas the fibroblastic-like cells of the outer AF produce a predominantly type I collagen-rich ECM. Simultaneously with vertebral body and AF morphogenesis, the notochord compressed at the vertebral segments, while expanding in between the vertebrae giving rise to the NP. Meanwhile, the CEP gradually forms with elongated, chondrogenic cells at the superficial zone of the developing vertebral bodies providing continuity with the inner AF. Postnatally, the AF, NP and CEP undergo further structural, compositional and mechanical maturation processes in order to fulfill the function of IVD in the spine.

IVD has to withstand large mechanical loads during three-dimensional spinal motion, therefore it is strongly exposed to pathological changes (Kim et al., 2012). Degeneration of the IVD is the most common spine condition leading to chronic back pain and physical disability in aging population (Buchbinder et al., 2013). The multiple interdependent risk factors include in particular environmental conditions, lifestyle and individual genetic predispositions (Sambrook et al., 1999; Battié and Videman, 2006; Kepler et al., 2013). Due to its complex and multifactorial pathogenesis, the degenerative mechanisms of the disease remain poorly understood. At early stages, the degeneration process of IVD involves a cascade of cellular changes that trigger structural reorganization and compositional changes of the extracellular matrix (ECM) resulting in the alteration of the tissue biomechanical properties and eventually functional impairment of the disc compartments (Lyons et al., 1981; Trout et al., 1982; Urban and McMullin, 1988; Pokharna and Phillips, 1998). The gradual ECM breakdown in IVD is hallmarked by progressing degradation of proteoglycans in NP and the adjacent AF, resulting in reduced water retention and inability to evenly distribute compressive forces to the VB (Lyons et al., 1966; Acaroglu et al., 1995; Sztrolovics et al., 1999). This in turn leads to circumferential and radial tears of the AF.

Aggrecan is the most abundant aggregating proteoglycan of the IVD tissues including the NP, the inner AF and the CEP. The 250 kDa aggrecan core protein is composed of three globular domains (G1, G2, G3) and intervening extended regions. In between the N-terminal G2 and the C-terminal G3 domains, the long glycosaminoglycan (GAG) attachment region is subdivided into segments rich in negatively charged keratin sulfate (KS) and chondroitin sulfate (CS1 and CS2) (Roughley et al., 2006). Aggrecan has the ability to form aggregates in association with hyaluronic acid and link protein through its N-terminal G1 domain (Watanabe and Yamada, 2002; Aspberg, 2012). The huge negative charge density carried by the sulfated GAGs endows the tissue with osmotic swelling capacity and thus resistance to compressive loads. Aggrecan function has been studied *in vivo* in animal models with spontaneous mutations in the aggrecan gene. Chick nanomelia, a lethal, recessively inherited short-limbed chondrodystrophy is caused by a premature stop codon in the CS2 coding region of exon 10 of the chicken aggrecan gene (Li et al., 1993; Primorac et al., 1994). In the naturally occurring cartilage matrix deficiency (*cmd*) mouse model, a 7 base pair deletion in exon 5 of the murine *Acan* gene results in premature translation termination, with the potential formation of a truncated protein consisting of a partial G1 domain (Watanabe et al., 1994). *Acan* ^*cmd/cmd*^ mutant mice are perinatally lethal and develop disproportionate dwarfism with cleft palate and abnormal endochondral bone formation with disorganized growth plate. Heterozygous *cmd* mice display proportionate dwarfism postnatally and late onset spine misalignment due to cervical lordosis and thoraco-lumbar kyphosis (Watanabe et al., 1997). The related *cmd* ^*bc*^ mutation is caused by the loss of exons 2-18, and *cmd* ^*bc*^ */cmd* ^*bc*^ mice show a *cmd*-like phenotype with severe defects in the skeletal elements of the spine, rib cage and limbs accompanied by the complete disruption of the normal cytoarchitecture of the growth plate (Lauing et al., 2014). In cattle, dexter bulldog dwarfism develops as a result of two causative gene mutations in exon 11 (BD1) and exon 1 (BD2) (Cavanagh et al., 2007). Affected homozygote animals are characterized by abortion, shortened limbs, vertebral column and ribs, pronounced vertebral platyspondyly and abdominal hernia. Heterozygous mutants are less affected with mild dwarfism, mild vertebral body irregularity and posterior wedging of vertebrae (Harper et al., 1998). In human, a series of allelic *ACAN* mutations are responsible for a broad range of non-lethal skeletal dysplasias affecting bone development and cartilage growth (Stattin et al., 2008; Tompson et al., 2009; Nilsson et al., 2014; Gibson and Briggs, 2016; Stattin et al., 2022).

In the present study, we report the generation of an insertional *Acan* mutant mouse line and describe the consequences of ACAN deficiency on vertebral column and intervertebral disc development. Mice homozygous for an insertion in exon 5 (*Acan* ^*iE5/iE5*^) die shortly after birth and mimic the *cmd*/*cmd* phenotype with severe skeletal defects, including significant shortening of both the appendicular and axial skeleton. Detailed analysis of the developing spine revealed abnormal vertebral bodies and defects in IVD formation accompanied by marked changes in nanomechanical properties of the cartilaginous elements of the IVD.

## 2 Materials and methods

### 2.1 Generation of the insertional mutant *Acan* ^*iE5*^ mouse line

To generate a functional *Acan-*null mutant mice, genomic *Acan* clones were isolated from P1-derived artificial chromosome (PAC) library using a mouse *Acan*-specific cDNA probe. A 12.4 kb BamHI genomic fragment carrying exons 2-9 was subcloned and used to generate the targeting construct. A neomycin resistance cassette flanked with two *frt* sites and one *loxP* site was introduced into intron 2, and a single *loxP* site carrying an engineered NsiI site was inserted into exon 5 to disrupt the *Acan* open reading frame. More details of the cloning strategy are available on request. The targeting construct was electroporated into R1 embryonic stem (ES) cells and subjected to G418 selection. Positive, homologous recombinant ES cell clones identified by Southern blotting of NsiI-digested genomic DNA were injected into C57BL/6J blastocysts, and the obtained male chimeras were mated with C57BL/6J female mice. Homozygous mice were obtained by intercrossing of heterozygous mice. Genotyping was performed by PCR using the forward primer 5′-TGC​CTC​TGT​CTC​TTC​CTA​TGG-3′ and the reverse primer 5′-TCT​CAT​TGG​TGT​CCC​GGA​TTC-3′ resulting in a 191 bp wild type and a 311 bp mutant band. Mice were handled and housed according to the federal institutional guidelines for the care and use of laboratory animals, approved by the Central Animal Facility of the LMU Munich and the government of Upper Bavaria (Application number: 55.2-1-54-2532-15-2016).

### 2.2 Western blot

Cartilage tissues dissected from the developing knee of wild type (WT), heterozygous (*Acan* ^*+/iE5*^) and homozygous mutant (*Acan* ^*iE5/iE5*^) animals at embryonic day 18.5 (E18.5) were pulverized in liquid N_2_. The samples were weighed and total protein extracted in 30 volumes of 4M guanidine hydrochloride solution with 50 mM sodium acetate, 0.1M ε-aminocaproic acid, 5 mM benzamidine, 5 mM N-ethylmaleimide (pH 5.8) for 48 h on an orbital shaker at 4°C. After ethanol precipitation, the pooled samples were dissolved in Tris-acetate buffer pH 8.0, and chondroitin sulfate chains digested by incubation with Chondroitinase ABC (Sigma-Aldrich, Taufkirchen, Germany), Chondroitinase ACII (Seikagaku, Tokyo, Japan) and Ovomucoid (Trypsin Inhibitor, Sigma T9253) protease inhibitor. Chondroitinase digested extracts were adjusted to NuPAGE LDS (Life Technologies, NP0007) loading buffer with dithiothreitol and heated at 70°C for 10 min. Pooled samples were loaded onto NuPAGE 4%–16% gels with antioxidant in the running buffer and separated at 200 V. One gel replicate was stained with BlueSilver colloidal Coomassie G250 (Fisher Scientific, BB 100-25) and served as loading control. The other replicate gels were electro-transferred onto PVDF membranes (Millipore, Immobilon IPFL 00010) in Novex transfer buffer (Life Technologies, NP0006). Blotted membranes were blocked in 5% dry milk in PBS-T (0.2% Tween-20 in PBS). Rabbit polyclonal antibody against the rat ACAN core protein was diluted 1:1000 in blocking solution and incubated O.N. at 4°C. Next day, after three washes in PBS-Tween, membranes were incubated for 1 h at room temperature with HRP-conjugated swine anti-rabbit IgG (Dako, P0217) in blocking solution. Afterwards, membranes were washed three time in PBS-T, and signals developed with Supersignal West Dura chemiluminiscent substrate (Thermo Scientific, Madison, WI, United States). Images were taken in a BioRad ChemiDoc MP device and processed with BioRad Image Lab software (BioRad, Berkeley, CA, United States).

### 2.3 Glycosaminoglycan assay

Cartilage tissues dissected from the developing knee of E18.5 embryos were weighted, snap frozen in liquid N_2_ and stored at −80°C until assayed. Tissues were digested in screw cap tubes for 6 h at 60°C with agitation in a 125 μg/mL papain solution containing 0.1 M sodium acetate pH 5.5, 5 mM EDTA, and 5 mM cysteine hydrochloride (all Sigma-Aldrich). Afterwards, samples were cooled down and centrifuged at 10,000 *g* for 10 min and supernatant collected. Sulphated glycosaminoglycans (sGAG) were quantified *via* the Blyscan sGAG assay kit (B1000; Biocolor Ltd., Carrickfergus, UK) according to manufacturer`s protocol. Optical density was measured at 650 nm on a Multiscan FC microtiter-plate reader (Thermo-Scientific) and plotted against a chondroitin 4-sulfate standard curve. Results are given as µg of sGAG per mg of wet tissue.

### 2.4 Skeletal staining

For skeletal staining, E18.5 embryos were de-skinned, eviscerated and fixed for 3 days in 95% ethanol following two additional days in acetone. The skeleton was stained with 0.6% Alcian Blue for cartilage and 0.02% Alizarin Red for bone elements (both Sigma-Aldrich) in 90% ethanol and 5% acetic acid for 3 days at 37°C with gentle shaking. Afterwards, specimens were cleared in gradually descending potassium hydroxide and ascending glycerol solutions (both Sigma-Aldrich) and finally preserved and imaged in 100% glycerol. Cervical spine from WT and *Acan* ^*iE5/iE5*^ mice were carefully dissected and imaged with a Stemi 1000 stereo microscope (Carl Zeiss, Jena, Germany).

### 2.5 Micro-computed tomography (µCT)

Embryos at E18.5 were scanned in air directly after sacrification using a ProCon X-Ray µCT device (CT-ALPHA, ProCon X-Ray GmbH, Sarstedt, Germany; DFG number: INST 409_211-1). Image acquisition was conducted with voxel size of 20 µm (70kv, 350µA, 0.5 mm aluminum filter, 0.24° rotation angle). The projection images were artifact corrected with the X-AID post-processing software (version 2022.7.0; MITOS GmbH, Munich, Germany). The 3D reconstruction and length measurements of the first lumbar (L1) vertebral body were performed using the Dragonfly 3D visualization and image analysis software (version 2022.1.0.1249; Object Research Systems Inc., Montreal, Canada).

### 2.6 Sample preparation

Whole embryos were isolated and directly immersed without fixation in Tissue Tek cryomedia (Sakura, Zoeterwoude, NL, United States). Samples were gradually frozen by positioning them on a chilled copper plate on dry ice. Cryosectioning was performed in the sagittal plane using a Microm HM500 cryostat (Thermo Scientific). Sections of spinal columns with a thickness of 9 µm were collected onto positively charged SuperFrost glass slides (Thermo Scientific) and kept at −20°C until use for histology, immunohistochemistry or AFM.

### 2.7 Histology and morphometric analysis

For histology, sections were thawed for 1 hour at RT, post-fixed in 4% PFA/PBS (Sigma-Aldrich) for 15 min at RT and rinsed three times in PBS. Hematoxylin and chromotrope 2R staining was performed to visualize tissue morphology. Slides were immersed in 1:2 Mayer´s hemalaum solution (Merck, Darmstadt, Germany) for 10 min and subsequently washed for 3 min with running tap water. Next, slides were passed through a graded series of 95% and 100% ethanol for 3 min each, followed by incubation with 0.1% chromotrope 2R (Sigma) in 90% ethanol and 0.1% acetic acid for 7 min. Afterwards, specimens were dehydrated in an ascending ethanol row, cleared twice in xylol and mounted with Roti-Histokit (Roth, Karlsruhe, Germany). Detection of sulphated glycosaminoglycans was carried out by Safranin-O staining. Briefly, slides were incubated with 0.5% Safranin-O (Sigma-Aldrich) in distilled water for 30 min, following two rinse in 95% and one rinse in 100% ethanol. Sections were mounted with Roti-Histokit (Roth). Von Kossa staining for calcium phosphate was used to visualize tissue mineralization. Slides were rinsed in dH_2_O and incubated for 15 min with 5% aqueous silver nitrate (AppliChem, Darmstadt, Germany) under the exposure of bright light. Afterwards, sections were washed in dH_2_O, running tap water and rinsed in dH_2_O, then counterstained with 1% neutral red/H_2_O for 2 min. Finally, sections were rapidly dehydrated, cleared in xylol and mounted with Roti-Histokit (Roth). High resolution overview pictures at 40x were taken with a PreciPoint M8 digital microscope (PreciPoint, Freising, Germany). Morphometric measurements were performed on E18.5 Safranin O pictures using the Polyline (for linear size of axis) and Polygon (for areas of IVD and VB) Annotation plug-ins of the ViewPoint Light Software (version: 1.0.0.9628) from PreciPoint. Cell density measurements of VB and ventral IA (vIA) at E14.5 and of NC, NP and vIA at E18.5 were performed on sections stained with the nuclear dye 4′,6-diamidino-2-phenylindole (DAPI) (Invitrogen, Carlsbad, CA, United States). Overlapped bright field and fluorescent photomicrographs were taken with an Axiocam MRm on an Axioskope 2 microscope (Carl Zeiss, Göttingen, Germany). Cell number and area measurements were analyzed using the Events and Draw Spline Contour tools of the ZEN 3.6 Lite software (Carl Zeiss), respectively. Results are shown as cells/mm^2^.

### 2.8 Immunohistochemistry

For immunohistochemistry, sections were thawed for 1 hour at RT, post-fixed in 4% PFA/PBS for 15 min at RT and rinsed three times in PBS. Next, endogenous peroxidases were quenched with a 0.3% H_2_O_2_/methanol solution for 20 min at RT. To facilitate antibody penetration, sections were digested with 2 mg/mL bovine testicular hyaluronidase/PBS (pH 5.0) (Sigma-Aldrich) at 37°C for 30 min. For type II collagen antibody staining, sections were blocked for 60 min at RT with M.O.M. blocking reagent and subsequently incubated with M.O.M. diluent for another 5 min (Vector Laboratories, Burlingame, CA, United States). For aggrecan antibody staining, sections were blocked with 1% bovine serum albumin (BSA)/PBS (Sigma-Aldrich) for 60 min at RT. For type I collagen antibody staining, sections were blocked with 0.25% goat serum/PBS for 60 min at RT. Slides were incubated overnight at 4°C with the following antibodies: primary mouse monoclonal type II collagen antibody (DSHB, Iowa, IA, United States; CIICI, 5 μg/mL) in M.O.M. diluent; rabbit polyclonal aggrecan antibody (AB 76-03 raised against the G1 domain of ACAN protein, 1:2000) in 1% BSA/PBS; rabbit polyclonal type I collagen antibody (Abcam, Cambridge, UK; ab34710, 1:200). Next morning, the appropriate biotinylated secondary antibody was applied to the tissue sections for 1 h at room temperature. The immunostaining was carried out using an avidin/biotin complex solution (Vectastain ABC Elite kit from Vector Laboratories) and the 3,3′-diaminobenzidine (Sigma) as chromogenic substrate. Finally, slides were mounted with Roti-Histokit (Roth). High resolution overview pictures at 40x were taken with a PreciPoint M8 digital microscope (PreciPoint).

### 2.9 Atomic force microscopy (AFM)

AFM-imaging and indentation-type AFM (IT-AFM) were performed with a NanoWizard I AFM (JPK Instruments, Berlin, Germany) combined with an inverted optical microscope (Axiovert 200, Carl Zeiss Micro Imaging GmbH, Göttingen, Germany), to ensure exact positioning of the AFM-tip onto the sample region of interest. This assembly was located on an active anti-vibration table (Micro 60, Halcyonics, Göttingen, Germany) inside a soundproof box. AFM was carried out using silicon nitride cantilevers (MLCT, Cantilever E, Bruker, Karlsruhe, Germany) with a nominal spring constant of 0.05–0.2 N/m and pyramidal tips with a nominal radius of 20 nm. For IT-AFM, the cantilever spring constant was determined applying a thermal noise method (Butt and Jaschke, 1995). For each cantilever, three calibrations were performed and the mean value was determined and used.

AFM-imaging was performed in contact mode in air. Images were recorded with a resolution of 512 × 512 pixels at line rates of 0.2–1.0 Hz. For a brief topography overview, images with a maximal scan size of 100 × 100 µm were obtained. The scan range was gradually scaled down to 3 × 3 μm, in order to enhance resolution. Recorded images were analyzed for collagen presence in the ECM of the selected IVD zones. Quantification of fibrils diameter was done using the open-source software Gwyddion 2.26. For each genotype and IVD zone, at least two high resolution 3 × 3 µm images obtained from 3 animals were evaluated.

IT-AFM measurements were carried out in PBS (pH 7.4). Elasticity measurements were conducted by a force mapping approach, recording 25 × 25 force-indentation curves within an area of 3 × 3 µm in the selected IVD zones (IM, PV, IA dorsal, IA ventral and CEP). Two different sections per animal with at least three different areas of interest in each IVD zone were investigated. All force-indentation curves were recorded with a constant vertical tip velocity of 15 μm/s. For each indentation point within the IVD zones, the Young’s modulus (E) was determined by fitting a modified Hertz model for a pyramidal indenter to the respective approach curve up to 500 nm of the maximal indentation depth using the JPK Data Processing software (Version 5.0.96, JPK Instruments). The contact point was determined manually for each force-distance curve. Final results are presented as Young´s moduli distribution in histograms rendering bimodal data distributions with two maxima (E1 and E2). In order to estimate both maxima in the histograms, a linear combination of two Gaussian distributions was fitted to the data of each histogram using Igor Pro software (Version 8.0.2.1, WaveMetrics, Portland, OR, United States).

### 2.10 Statistical analysis

Statistical analysis was performed with GraphPad Prism (San Diego, CA, United States). Datasets were tested for normal distribution and range of variances. Afterwards appropriate *t*-test or one-way ANOVA were run with the proper *post hoc* tests. Statistical significance was assumed at a *p*-value of ≤0.05. A minimum of 3 animals per genotype were used in each experiment.

## 3 Results

### 3.1 Generation and gross characterization of aggrecan insertional mutant mice

**FIGURE 1:**
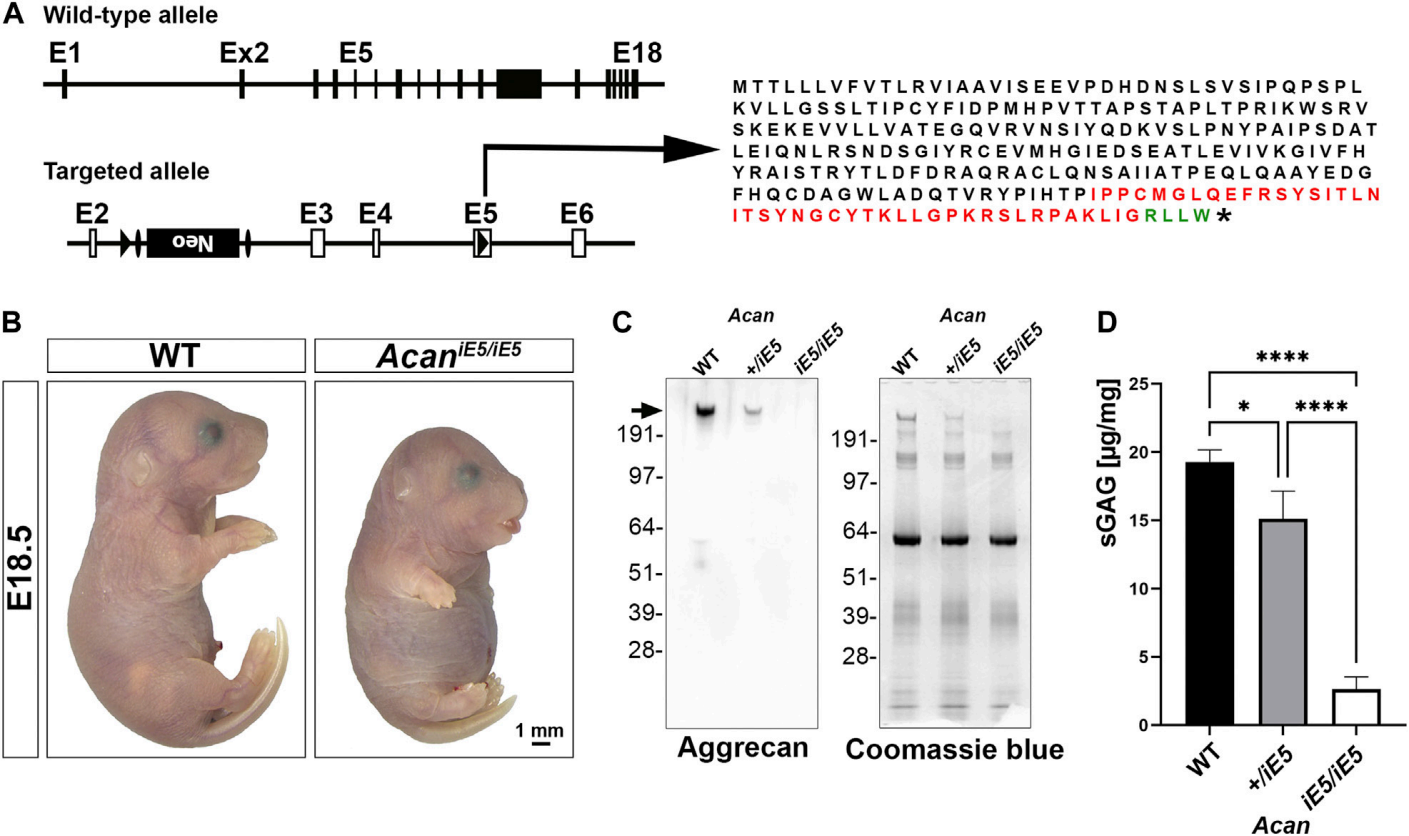
Generation of *Acan*-deficient mouse. **(A)** Targeted inactivation of the *Acan* gene. The *Acan* locus was targeted by introducing a *lox*P site into exon 5 and a *lox*P-*Frt*-*neo*-*Frt* cassette into intron 2. Insertion of *lox*P in exon 5 resulted in the translation into a 263 aa long truncated protein. Black, red and green colors represent amino acids of aggrecan, insertion sequence and non-aggrecan, respectively, before the stop codon (*). **(B)** Representative macrophotograph of WT and *Acan* ^*iE5/iE5*^ E18.5 embryos. Homozygous mutant mice die shortly after birth due to respiratory distress and exhibit abnormal phenotype characterized by severe skeletal malformations. **(C)** Western blot (left) against the full form of ACAN (arrow) demonstrates the complete absence of protein in the pooled cartilage tissue of the developing knee at E18.5 in *Acan* ^*iE5/iE5*^ mice, and about 50% reduction in *Acan* ^*+/iE5*^ mice. On the right, Coomassie blue staining shows equal protein loading of the gel. **(D)** 1, 9-dimethylmethylene blue assay showing the partial and almost complete loss of sGAG in respectively, *Acan* ^*+/iE5*^ and *Acan* ^*iE5/iE5*^ E18.5 embryos cartilage tissue (*n* = 3). Statistical significance calculated by one-way ANOVA with Tukey’s multiple comparison test, where *: *p* ≤ 0.05 and ****: *p* < 0.0001.

*Acan* mutant mice were generated by interrupting *Acan* expression in exon 5 (Figure 1A). Insertion of a *lox*P sequence disrupted the region of open reading frame encoding the N-terminal G1 domain and resulted in the translation of a hypothetic truncated protein of 263 amino acids with 216 amino acids representing the N-terminal part of the normal, full length mouse ACAN protein (2132 amino acids). Heterozygous mice are alive, breed normally and are indistinguishable from the wild type littermates (not shown). Homozygous mutant mice die at birth due to respiratory failure and are characterized by severe proportionate dwarfism, distended abdomen, short snout and cleft palate that phenocopy the naturally occurring *cmd* mice (Rittenhouse et al., 1978) (Figure 1B). The functional null mutation was confirmed at the protein level in cartilage tissue extracts from the developing hindlimbs of E18.5 embryos. Western blotting with a rabbit polyclonal antibody against total ACAN demonstrated the absence of aggrecan protein in homozygous mutant cartilage and an approximately halved expression in heterozygous mice compared to the wild type (Figure 1C). As aggrecan is the main contributor of sGAG in cartilaginous-like tissues, we quantified sGAG levels in cartilage tissues from the wild type, heterozygous and homozygous mutant animals. A significant decrease in sGAG concentration of 86.35% and 21.59% was recorded in *Acan* ^*iE5/iE5*^ and *Acan* ^*+/iE5*^ animals, respectively, compared to their wild type littermates (WT: 19.27 ± 0.89 μg/mg; *Acan* ^*+/iE5*^: 15.11 ± 2.04 μg/mg; *Acan* ^*iE5/iE5*^: 2.63 ± 0.90 μg/mg; WT vs. *Acan* ^*+/−*^: *p* = 0.024; WT and *Acan* ^*+/iE5*^ vs. *Acan* ^*iE5/iE5*^: *p* < 0.0001) (Figure 1D).

### 3.2 Disruption of the aggrecan gene leads to severe skeletal malformations

**FIGURE 2:**
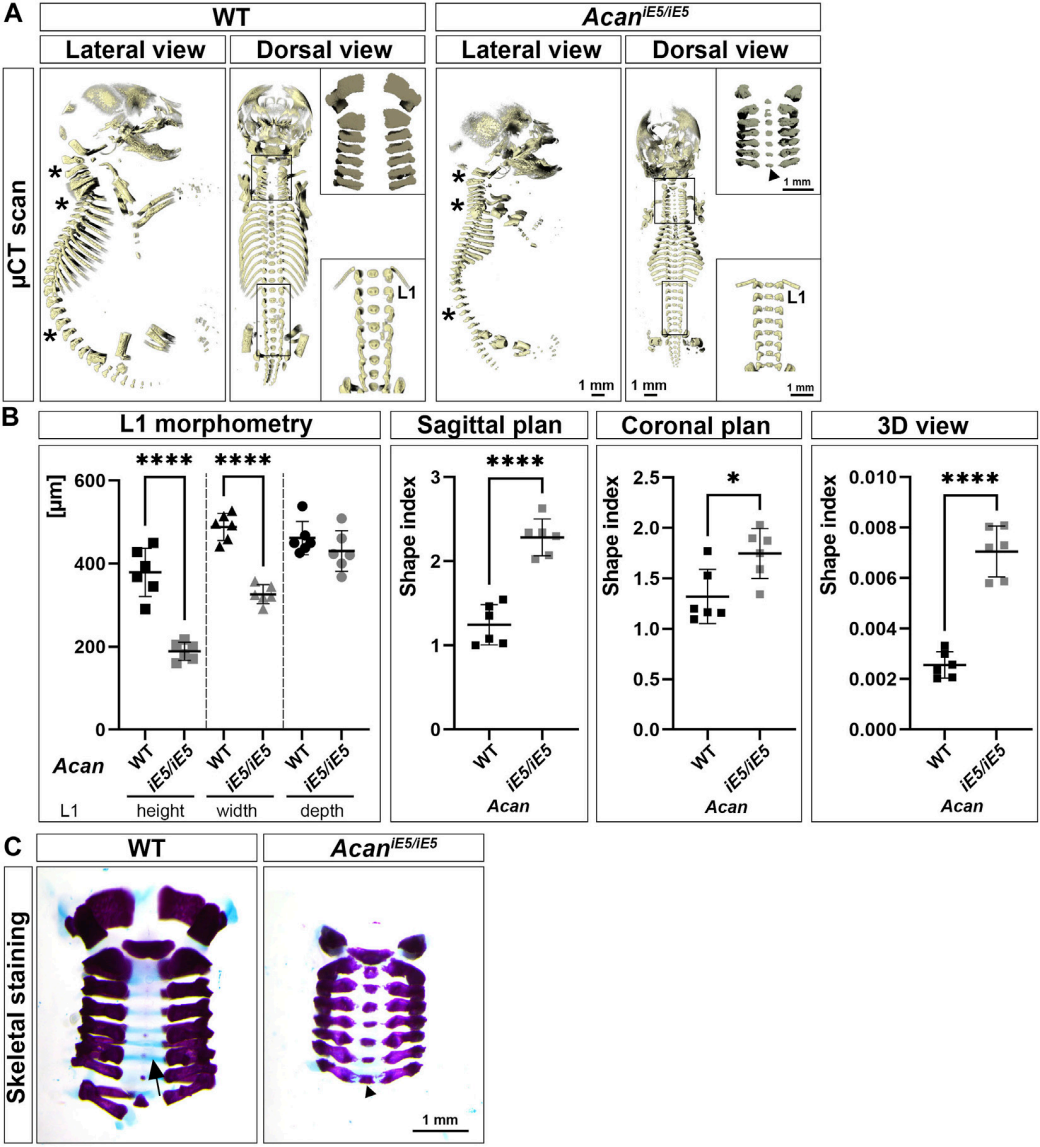
Characterization of skeletal abnormalities of aggrecan-deficient mice. **(A)** Representative 3D reconstruction of µ-CT scans of the sagittal and coronal views of the skeleton of E18.5 WT and *Acan* ^*iE5/iE5*^ embryos. Asterisks depict the differences in spine alignment in between the genotypes. Upper and bottom inserts show the enlarged cervical and lumbar spinal regions, respectively. Arrowhead indicates premature ossification of the cervical vertebral bodies in the mutant. **(B)** Morphometric analysis of the L1 vertebra. 2D shape index of coronal and sagittal views and 3D shape index. Values represents the mean ± STD. Statistical significance calculated by unpaired *t*-test test, where *: *p* ≤ 0.05 and ****: *p* < 0.0001. **(C)** Whole-mount skeletal staining of the isolated cervical region of E18.5 embryos, demonstrating the absence of staining for sGAG in *Acan* ^*iE5/iE5*^ mice and the concomitant accelerated calcification of the VB in mutant mice (arrowhead). Arrow shows the Alcian blue positive vertebral bodies in the wild type animals.

Confirming the visual inspection, 3D reconstruction of µ-CT images at E18.5 demonstrated a severe reduction in the length of the axial and appendicular skeletal elements of ACAN-deficient mice compared to their control littermates (Figure 2A). In addition, lateral view µ-CT images revealed changes in the spine curvature of the mutant mice, i.e., abnormal positioning of the atlas and axis, angle reduction of the cervical and thoracic spine sections, and straightening of the lumbar part in mutant mice (asterisks in Figure 2A, lateral view). Morphometric measurement of the first lumbar vertebra (L1) showed significant, 1.97-fold height (rostral-caudal; WT: 379.2 ± 57.76 µm vs. *Acan* ^*iE5/iE5*^: 189.1 ± 21.74 µm; *p* < 0.0001) and 1.5-fold width (medial-lateral; WT: 488.4 ± 32.79 µm vs. *Acan* ^*iE5/iE5*^: 326.3 ± 22.76 µm; *p* < 0.0001) reduction in mutant animals compared to wild type controls (Figure 2B). Ventral-dorsal depth of L1 of WT and *Acan* ^*iE5/iE5*^ animals was in a similar range (*p* = 0.25). These differences reflect the altered geometry of the vertebral bodies in mutant mice. Calculation of shape indexes on sagittal (WT: 1.25 ± 0.24 vs. *Acan* ^*iE5/iE5*^: 2.29 ± 0.22; *p* < 0.0001), coronal (WT: 1.32 ± 0.27 vs. *Acan* ^*iE5/iE5*^: 1.75 ± 0.25; *p* = 0.0167) and 3D (WT: 0.0026 ± 0.00052 vs. *Acan* ^*iE5/iE5*^: 0.0071 ± 0.0001; *p* < 0.0001) views of L1, further demonstrate the squeezed phenotype of vertebrae in mutant mice. Heterozygous mice displayed similar L1 morphometry to the wild type littermates (data not shown). Alcian blue in the skeletal staining of E18.5 embryos, depicted the cartilaginous center of cervical vertebrae (blue staining, arrow in Figure 2C), whereas mutants exhibited little if any blue staining indicating the lack of sGAG (Figure 2C). Both µCT scans and Alizarin red in the whole-mount skeletal staining demonstrated accelerated calcification of the central part of the cervical vertebral bodies in *Acan* ^*iE5/iE5*^ mice (Figure 2A, upper insert, dorsal view; and Figure 2C).

### 3.3 *Acan* ^*iE5/iE5*^ mice present collapsed ECM with high cellular density and altered IVD morphometry

**FIGURE 3:**
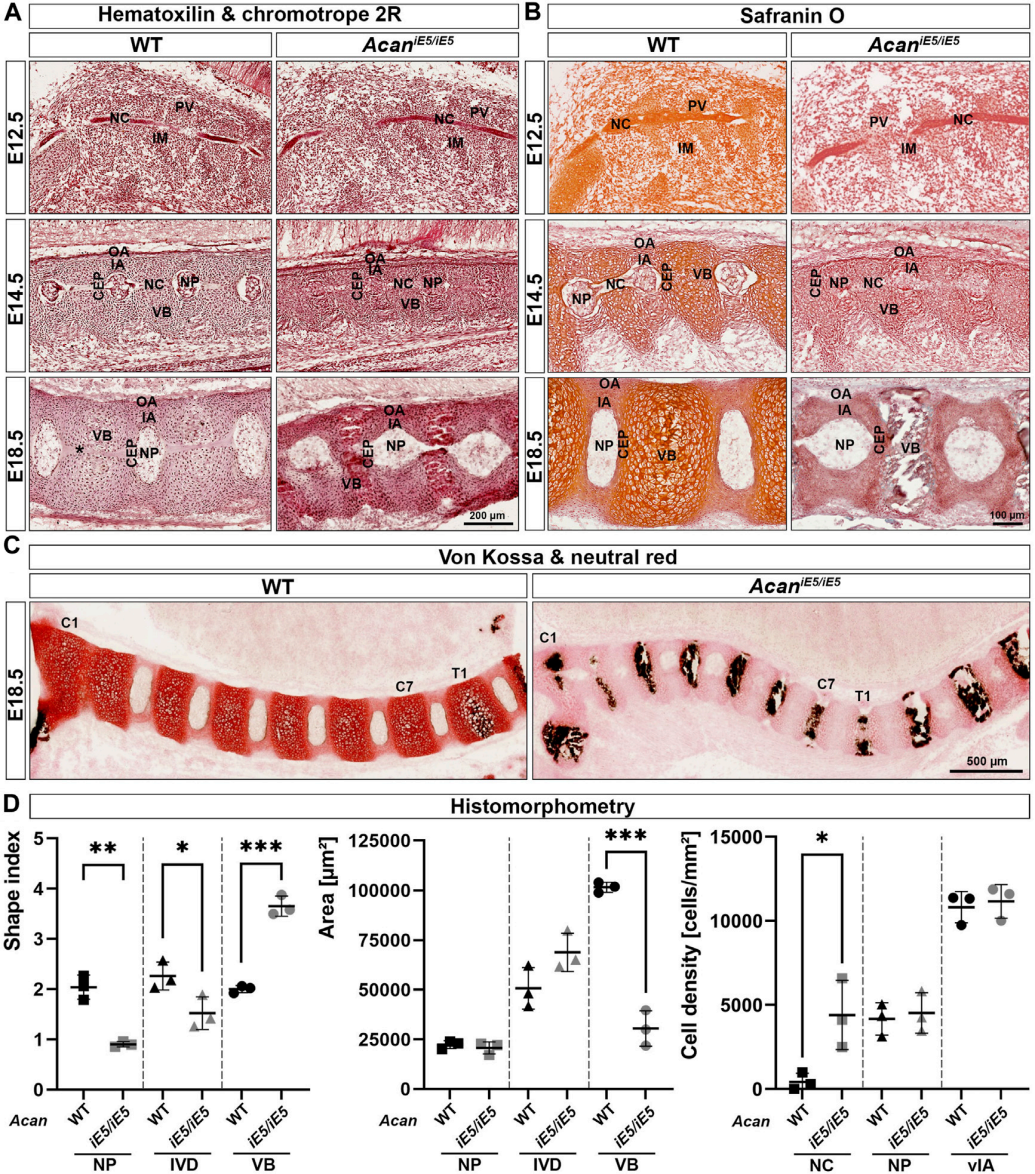
**(A)** Representative images of Hematoxylin and Chromotrope 2R stained cervical vertebral columns in *Acan* ^*iE5/iE5*^ and WT mice at E12.5, E14.5 and E18.5. Scale bar, 200 µm. **(B)** Safranin O staining showing that proteoglycan deposition in the ECM of the spinal column of *Acan* ^*iE5/iE5*^ mice is negligibly low. Scale bar, 100 µm. **(C)** Von Kossa and neutral red staining demonstrating the premature mineralization of the cervical (C1-C7) VBs in mutant mice. Wild type shows black calcium phosphate deposits only from the level of the first thoracic (T1) vertebra. Scale bar, 500 µm. Top-bottom and left-right of every picture correspond to the dorsal-ventral and rostral-caudal anatomical orientations, respectively. **(D)** Histomorphometric analysis of shape index, area and cell density of the depicted spine compartments of E18.5 animals. Abbreviations: NC, notochord; IM, intervertebral mesenchyme; PV, prevertebrae; NP, nucleus pulposus; VB, vertebral body; CEP, cartilage end plate; IA, inner annulus fibrosus; vIA, ventral IA; OA, outer annulus fibrosus. Data represent the mean ± STD. Statistical significance calculated by unpaired *t*-test, where *: *p* ≤ 0.05; **: *p* < 0.01 and ***: *p* < 0.001.

To further investigate the abnormalities of the axial skeleton in the aggrecan-deficient mice, histological analysis of vertebral columns at E12.5, E14.5 and E18.5 developmental stages were performed. Hematoxylin-Chromotrope 2R and Safranin O staining of cervical spine sections (Figures 3A, B) at E12.5 revealed areas of metameric patterns with non-uniformly condensed zones. Both WT and *Acan* ^*iE5/iE5*^ animals showed typical formation of less condensed, pre-cartilaginous areas of the prevertebrae (PV) and more condensed regions of the intervertebral mesenchyme (IM). In both genotypes, the notochord (NC) was a continuous structure of spherical cells enclosed in a sheet, extending rostro-caudally in the midline of the surrounding PVs and IM. By E14.5, the mesenchymal tissue surrounding the NC had progressively differentiated to form compartments of the IVDs and the vertebral bodies (VBs) in both mutant and control mice. The dense IM differentiated into the inner and outer AF (IA and OA), whereas PVs enlarged and developed into VBs. Furthermore, the NC between the VBs was dilated and formed the nucleus pulposus (NP), the central part of the IVD. Although all compartments of the IVDs and the adjacent VBs were formed in both genotypes, the mutant embryos displayed marked abnormalities. The vertebral bodies and the cartilaginous IA appeared smaller (VB: WT 54004 ± 518.4 μm^2^, *Acan^iE5/iE5^*: 39028 ± 2890 μm^2^, *p* = 0.0009; IA: WT 7208 ± 1191 μm^2^, *Acan^iE5/iE5^*: 3614 ± 85.4 μm^2^, *p* = 0.0065) with high cellular density (VB: WT 8422 ± 401.4 cells/mm^2^, *Acan^iE5/iE5^*: 10478 ± 607.7 cells/mm^2^, *p* = 0.0081; IA: WT 11972 ± 2074 cells/mm^2^, *Acan^iE5/iE5^*: 16572 ± 4143 cells/mm^2^, *p* = 0.1606) and collapsed ECM compared to control. The concentric organization of elongated cells, characteristic of control IA, was largely diminished in the mutant. In *Acan* ^*iE5/iE5*^ mice the developing NPs were smaller between the vertebrae, the NP cells were less vacuolated and did not detach from the vertebral segments of the notochord than in wild type embryos. At E18.5, these abnormalities became even more apparent. While the NPs in controls were laterally expanded and disc-shaped, the mutant NPs remained rounded. Importantly, the cells did not fully vanish from the vertebral segments of the NC of the mutants as can be observed in the control animals (Figure 3A). The *Acan* ^*iE5/iE5*^ cervical VBs were compressed and their central part was completely mineralized as demonstrated by the von Kossa positive staining in Figure 3C. In contrast, calcium deposits were only demonstrated at and below the first thoracic vertebra (T1) in wild type animals. Furthermore, in mutant tissues the growth plate was not visible, and the CEPs appeared thinner and disorganized compared to the control tissues. In addition, the circumferential organization of the IA cells were largely absent in the mutant. As expected, the vertebral column of *Acan* ^*iE5/iE5*^ mice was negligibly positive for Safranin-O staining throughout embryogenesis (Figure 3B). In support of this finding, we also detected fainter staining using the cationic dye neutral red in mutant cartilaginous tissues at E18.5 demonstrating greatly depleted proteoglycan content (Figure 3C).

Morphological differences and cellular phenotype between genotypes were quantitatively evaluated in mice at E18.5 by determining the shape index, the area and the cell density of different cervical spine compartments (Figure 3D). In mutants, the shape index of all measured cervical spine compartments changed significantly compared to control littermates. The shape index of NP decreased from 2.04 ± 0.24 in WT to 0.90 ± 0.05 in *Acan* ^*iE5/iE5*^ mice (0.44-fold; *p* = 0.0013), reflecting the change of NP geometry from discoid to nearly rounded. Similarly, the overall IVD shape index decreased from 2.26 ± 2.79 in WT to 1.52 ± 0.33 in *Acan* ^*iE5/iE5*^ animals (0.67-fold; *p* = 0.0404). In contrast, the shape index of mutant VBs was 1.83-fold higher than in control (*p* = 0.0002) reaching a value of 3.65 ± 0.20, compared to the wild type tissues, which had a shape index of 2.00 ± 0.07. Next, we compared the size of the spine compartments of the different genotypes. The area of NP remained almost unchanged in WT and *Acan* ^*iE5/iE5*^ mice (WT: 22313 ± 1982 μm^2^ and *Acan* ^*iE5/iE5*^: 20675 ± 3059 μm^2^; *p* = 0.4799). The area of mutants IVD increased 1.36-fold compared to control tissues, although did not reach statistical significance (WT: 50656 ± 10456 μm^2^; *Acan* ^*iE5/iE5*^: 68788 ± 9636 μm^2^; *p* = 0.0918). Finally, as indicated by the µ-CT scans, the area of *Acan* ^*iE5/iE5*^ VB decreased significantly in comparison to WT animals (WT: 101470 ± 2571 μm^2^; *Acan* ^*iE5/iE5*^: 30459 ± 8914 μm^2^; 0.30-fold; *p* = 0.0002). Cell density measurements at E18.5 revealed a cell-populated notochord at the vertebral segments of the mutant mice, while the vertebral notochord was almost completely cell-free in wild types (WT: 423 ± 502.7 cells/mm^2^; *Acan* ^*iE5/iE5*^: 4308 ± 2057 cells/mm^2^; *p* = 0.0313). Cellular density in the NB and the ventral IA was not significantly different between genotypes.

### 3.4 Lack of aggrecan does not impairs the deposition of type II and I collagens

**FIGURE 4:**
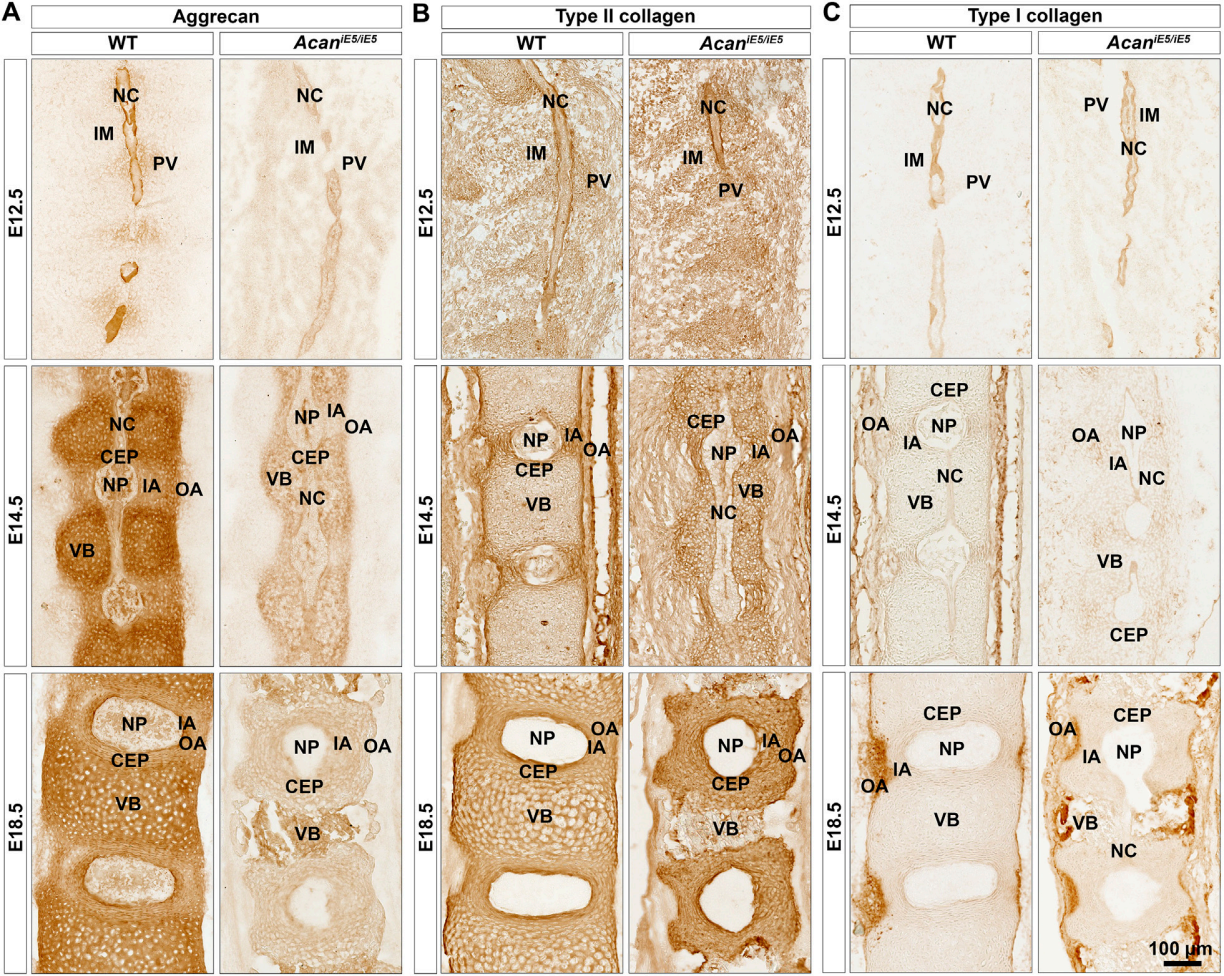
Immunohistochemistry against aggrecan **(A)**, type II collagen **(B)** and type I collagen **(C)** on the cervical portion of vertebral columns in WT and *Acan* ^*iE5/iE5*^ mice at E12.5, E14.5 and E18.5. **(A)** Representative pictures demonstrating the expression pattern of aggrecan in the developing spine tissues in wild type, and its almost total absence in mutant mice. **(B)** Type II collagen deposition was not affected in *Acan* ^*iE5/iE5*^ mice. **(C)** Type I collagen expression was comparable between the genotypes. Bar, 100 µm. The mid-sagittal sections in all panels are presented at the same scale. Top-bottom and left-right of every picture correspond to the rostral-caudal and ventral-dorsal anatomical orientations, respectively. Abbreviations: NC, notochord; IM, intervertebral mesenchyme; PV, prevertebrae; NP, nucleus pulposus; VB, vertebral body; CEP, cartilage end plate; IA, inner annulus fibrosus; OA, outer annulus fibrosus.

Immunohistochemical analysis in wild type mice using a G1 specific antibody confirmed a mild deposition of aggrecan in the prevertebral mesenchyme and the notochord, and its strong expression in the NC sheath at E12.5 (Figure 4A). During normal spine development in E14.5 and E18.5 control embryos, ACAN expression was detectable in multiple tissues. Aggrecan strongly stained in the cartilaginous VBs and moderately in the CEP, NP and the IA (Figure 4A). Unexpectedly, a very faint ACAN signal was also observed in *Acan* ^*iE5/iE5*^ mice along the entire vertebral column development (Figure 4A) which may suggest that the truncated protein is detectable in the mutant tissue. The deposition of collagens and proteoglycans concomitantly occurs during the embryogenesis of cartilaginous tissues. Type II collagen was comparably localized in wild type and *Acan* ^*iE5/iE5*^ mice throughout the development (Figure 4B). At E12.5, strong type II collagen immunoreactivity was demonstrated in the notochordal sheath and weak signal was observed in the PVs. At E14.5, the immunostaining for collagen type II in the cervical vertebral columns revealed positive staining in the IA, the notochordal sheath, the CEP and the VB, and the lack of signal in the OA. At E18.5, strong signal was detected in the IA, CEP and VB of both genotypes. Strong collagen I deposition was observed in the notochordal sheath at E12, and in the OA at E14.5 and E18.5 in both wild type and *Acan* ^*iE5/iE5*^ cervical spine (Figure 4C). Interestingly, weak collagen I immunoreactivity was observed in the IA at E14.5 in both genotypes. There was no obvious difference in the collagen I staining pattern between wild type and *Acan* ^*iE5/iE5*^ cervical spine throughout the development.

### 3.5 The ECM of *Acan* ^*iE5/iE5*^ mice is characterized by thinner collagen fibrils

**FIGURE 5:**
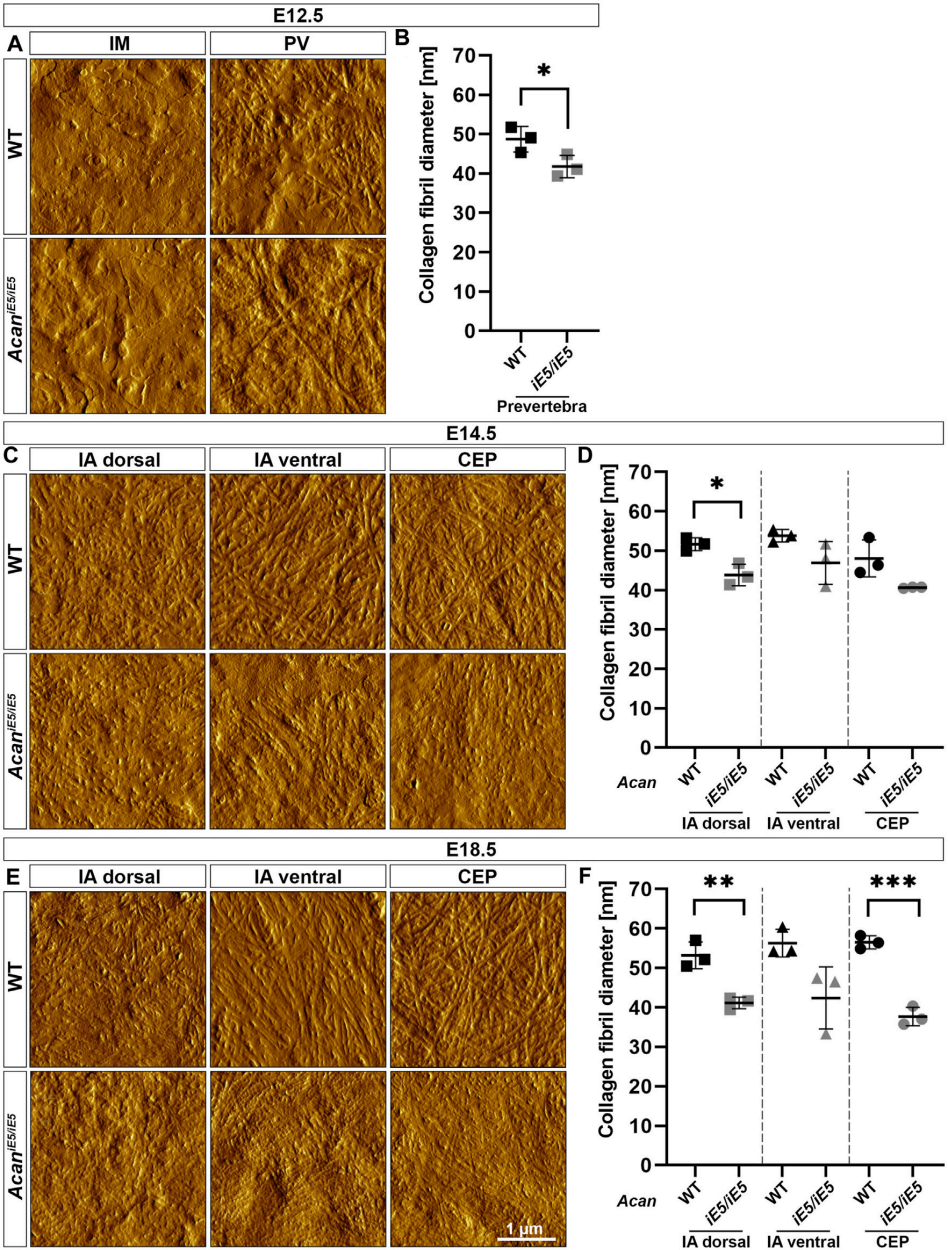
Characterization of the collagen network in the developing IVDs of the cervical spine in WT and *Acan* ^*iE5/iE5*^ mice. **(A, C, E)** High-resolution (3 × 3 µm) AFM images of the ECM were acquired in the IM and PV at E12.5, and in the IA dorsal, IA ventral and CEP at E14.5 and E18.5. Scale bar, 1 µm. **(B, D, F)** Quantification of collagen fibril diameter. Bars represent the mean ± STD. At least one AFM image and 50 fibrils were analyzed per independent mouse, *n* = 3. Statistical significance calculated by *t*-test where *p < 0.05; **p < 0.01; ***p < 0.001.

AFM high-resolution imaging was implemented to resolve collagen fibrils organization of the developing IVD compartments of the cervical spine. At E12.5, in both control and *Acan* ^*iE5/iE5*^ animals, a network of long, irregularly interconnected thin collagen fibrils was visible within the ECM of the PV, whereas in IM regions a rather homogeneous matrix without clearly discernable fibrils was observed (Figure 5A). Fibril diameter measurements revealed a significantly reduced fibril thickness (0.86-fold; *p* = 0.049) in the PV regions of *Acan* ^*iE5/iE5*^ mice: the mean fibril diameter of 41.78 ± 2.83 nm compared to 48.73 ± 3.24 nm in control mice (Figure 5B). At E14.5, AFM topographic images showed differences in ECM organization not only between the genotypes but also among the three distinct IVD regions (IA dorsal, IA ventral and CEP) (Figure 5C). In WT, regions of the dorsal and ventral IA exhibited collagen fibrils that were loosely packed into randomly oriented and irregularly distributed thick bundles. In contrast, collagen fibrils density and the degree of organization within the ECM were higher in the wild type CEP compared to the adjacent IA. In *Acan* ^*iE5/iE5*^ mice, collagen fibers of variable length in all three IVD zones showed a more compact collagen network compared to wild type. The means of fibril diameter in WT were 51.65 ± 1.63 nm (IA dorsal), 53.79 ± 1.56 nm (IA ventral) and 48.06 ± 4.68 nm (CEP) (Figure 5D). In *Acan* ^*iE5/iE5*^ mice, fiber diameter was reduced by 0.85-fold in both IA dorsal (*p* = 0.013) and CEP, and by 0.87-fold in IA ventral compared to control animals (Figure 5D). At E18.5, AFM vertical deflection images of wild type IVD revealed a very dense and well-organized cross-banded collagen network with thick bundles in all three compartments, whereas the collagen network in mutant IVD appeared further collapsed with thinner and denser interwoven fibrils (Figure 5E). During IVD development in control mice, the mean diameter of collagen fibrils increased gradually, reaching the largest diameter in IA ventral (56.3 ± 3.51 nm) and CEP (56.5 ± 1.67 nm) followed by IA dorsal (53.2 ± 3.39 nm) at E18.5 (Figure 5F). Compared to control mice, mutant E18.5 animals showed mean collagen fiber thickness reduced by 0.77-fold (*p* = 0.005), 0.75-fold and 0.67-fold (*p* = 0.0004) in IA dorsal, IA ventral and CEP, respectively.

### 3.6 Loss of aggrecan leads to stiffening of the ECM in all IVD compartments

**FIGURE 6:**
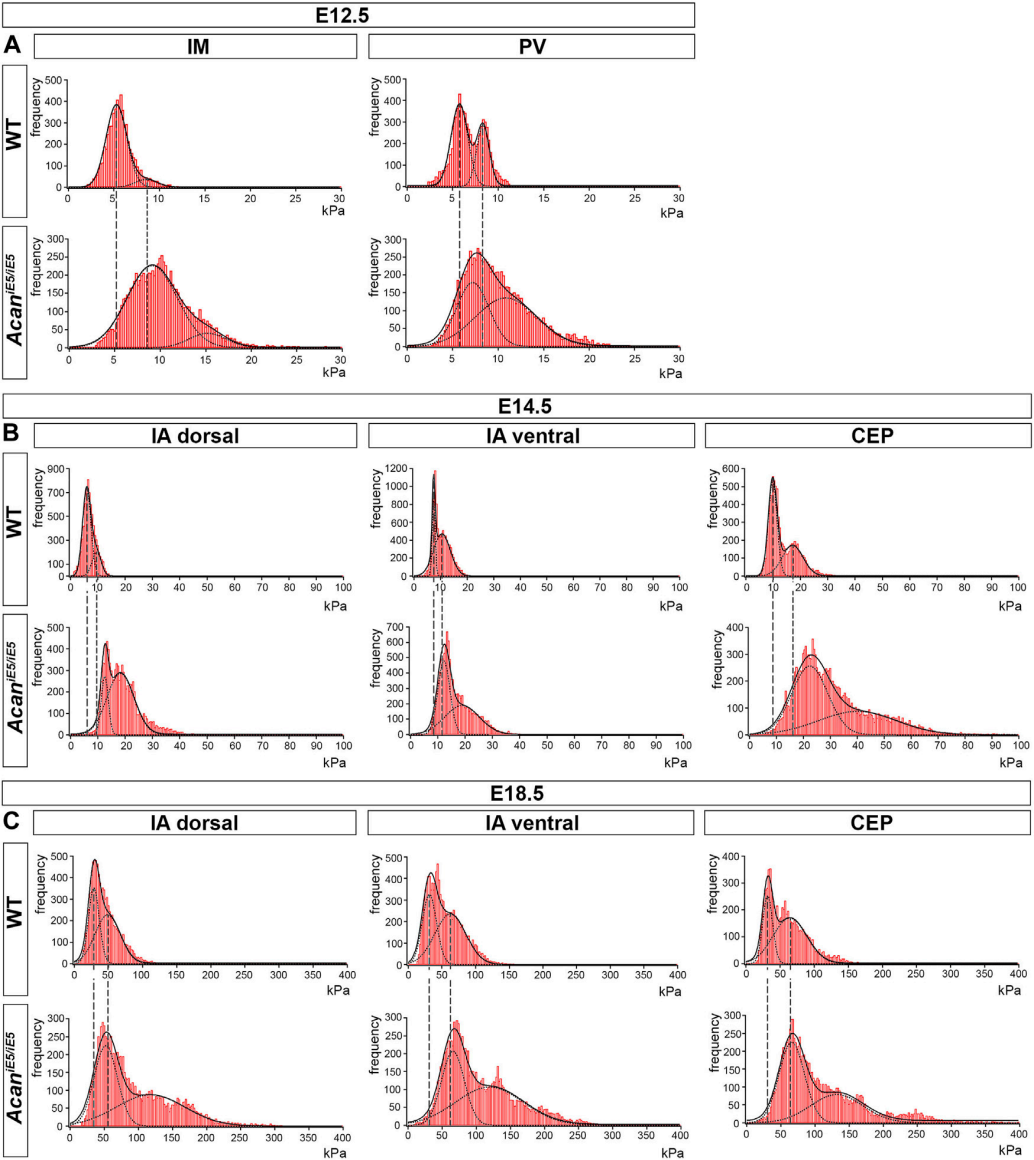
Stiffness distribution of the ECM within the IM and PV at E12.5 **(A)**, and the IA dorsal, IA ventral and CEP at E14.5 and E18.5 **(B,C)** determined by IT-AFM. Each graph shows data pooled from three independent animals. Solid lines represent a linear combination of two Gaussian functions while the dashed lines denote individual Gaussian distributions representing the softer proteoglycan and stiffer collagenous phases of cartilaginous ECM. Vertical dashed lines indicate shifts of proteoglycan and collagen stiffness in the mutants.

In order to assess the impact of ACAN deficiency on the biomechanical properties of the different IVD zones, we performed a nano-scale IT-AFM analysis during the development of the cervical spine. In both WT and *Acan* ^*iE5/iE5*^ mice, histograms of the Young’s moduli (Figure 6) demonstrated a bimodal stiffness distribution in all compartments of the developing IVD, with characteristic peaks of the two Gaussian fits indicating the softer intrafibrillar ECM, usually dominated with proteoglycans, and the stiffer collagen network (Loparic et al., 2010; Prein et al., 2016; Alberton et al., 2019; Fleischhauer et al., 2022). The lack of functional ACAN in the mutant mice resulted in a marked shift towards higher stiffness values (Figure 6), indicating that aggrecan is the determining proteoglycan for the soft moiety of the cartilaginous tissues.

Quantitative and statistical analysis of the lower and higher peaks (E1 and E2) confirmed the importance of ACAN in IVD tissue biomechanics (Table 1). At E12.5, the stiffness measurements in mutant IM zones revealed significantly increased stiffness mean values by 1.72-fold for E1 (WT: 5.23 ± 0.17 kPa vs. *Acan* ^*iE5/iE5*^: 8.997 ± 0.893 kPa) and by 1.78-fold for E2 (WT: 8.65 ± 1.14 kPa vs. *Acan* ^*iE5/iE5*^: 15.38 ± 0.86 kPa) compared to wild type. Similarly, the mutant PVs showed significantly higher stiffness mean values of E1 (7.34 ± 0.90 kPa) and E2 (11.49 ± 0.63 kPa) compared with wild type (E1 = 5.38 ± 0.80 kPa, E2 = 7.71 ± 0.82 kPa) (E1: 1.37-fold increase and E2: 1.49-fold increase).

**TABLE 1:** Summary of the calculated mean elastic moduli at the E12.5, E14.5 and E18.5 in the developing IVD compartments.

E12.5			
		WT	*Acan ^iE5/iE5^*
**IM**	Mean E1	5.225 ± 0.166	8.997 ± 0.893
	*p* -value	*p* = 0.002 (**)	
	Mean E2	8.647 ± 1.140	15.380 ± 0.856
	*p* -value	*p* = 0.001 (**)	
	Range min	2.277 ± 1.237	2.921 ± 0.765
	Range max	11.974 ± 1.134	28.500 ± 2.141
**PV**	Mean E1	5.377 ± 0.798	7.343 ± 0.904
	*p* -value	*p* =0.048 (*)	
	Mean E2	7.713 ± 0.823	11.490 ± 0.627
	*p* -value	*p* =0.003 (**)	
	Range min	2.300 ± 1.649	3.015 ± 0.974
	Range max	14.939 ± 0.366	27.702 ± 2.310

E14.5			
		WT	*Acan ^iE5/iE5^*
**IA dorsal**	Mean E1	6.138 ± 1.310	12.270 ± 0.402
	*p* -value	*p* =0.002 (**)	
	Mean E2	9.633 ± 1.015	18.910 ± 3.358
	*p* -value	*p* =0.010 (*)	
	Range min	2.261 ± 1.326	5.559 ± 2.987
	Range max	18.142 ± 1.396	40.099 ± 8.754
**IA ventral**	Mean E1	8.623 ± 1.874	12.450 ± 0.371
	*p* -value	*p* =0.026 (*)	
	Mean E2	11.100 ± 1.760	21.460 ± 4.271
	*p* -value	*p* =0.017 (*)	
	Range min	3.406 ± 2.375	6.710 ± 1.099
	Range max	21.824 ± 2,037	42.092 ± 8.364
**CEP**	Mean E1	10.230 ± 1.092	22.630 ± 3.743
	*p* -value	*p* =0.005 (**)	
	Mean E2	20.230 ± 2.721	40.180 ± 7.988
	*p* -value	*p* =0.015 (*)	
	Range min	3.610 ± 0.720	8.595 ± 4.779
	Range max	38.236 ± 13.147	84.295 ± 15.564

E18.5			
		WT	*Acan ^iE5/iE5^*
**IA dorsal**	Mean E1	27.820 ± 0.590	54.670 ± 11.620
	*p* -value	*p* =0.057 (ns)	
	Mean E2	51.880 ± 4.945	137.400 ± 11.430
	*p* -value	*p* =0.0003 (***)	
	Range min	13.059 ± 6.808	18.191 ± 7.204
	Range max	115.577 ± 13.067	351.685 ± 16.253
**IA ventral**	Mean E1	33.620 ± 3.624	62.540 ± 6.609
	*p* -value	*p* =0.003 (**)	
	Mean E2	69.250 ± 2.592	138.100 ± 11.860
	*p* -value	*p* =0.008 (**)	
	Range min	14.039 ± 2.195	18.872 ± 7.571
	Range max	155.846 ± 30.466	369.231 ± 32.743
**CEP**	Mean E1	35.470 ± 9.278	63.590 ± 6.697
	*p* -value	*p* =0.013 (*)	
	Mean E2	70.820 ± 7.021	139.200 ± 4.928
	*p* -value	*p* =0.0002 (***)	
	Range min	15.672 ± 4.729	22.745 ± 10.711
	Range max	178.599 ± 31.496	392.882 ± 18.974

Statistical significance between genotypes was calculated by *t*-test, with *p < 0.05; **p < 0.01; and ***p < 0.001.

In wild type at E14.5, the dorsal IA exhibited the lowest stiffness mean value of E1:6.14 ± 1.31 kPa, while the ventral IA and the CEP showed slightly higher E1 values of 8.62 ± 1.87 kPa and 10.23 ± 1.09 kPa, respectively. Regarding E2, the dorsal and ventral IA showed comparable mean values (9.63 ± 1.01 kPa and 11.10 ± 10 kPa, respectively), whereas the CEP region appeared to be about twice as stiff with mean E2 of 20.23 ± 2.72 kPa. When comparing *Acan* ^*iE5/iE5*^ mice to control littermates, stiffness mean values were significantly increased in the dorsal IA (E1: 2.00-fold, E2: 1.96-fold), ventral IA (E1: 1.44-fold, E2: 1.93-fold) and CEP (E1: 2.21-fold; E2: 1.99-fold) (Table 1).

In E18.5 control IVD, the recorded individual stiffness mean values were markedly increased compared to the corresponding stiffness at E14.5. E1 reached 27.82 ± 0.59 kPa in dorsal IA, 33.62 ± 3.62 kPa in ventral IA and 35.47 ± 9.28 kPa in CEP (Table 1). The E2 values were approximately twice as high as the corresponding E1 values (E2 in dorsal IA: 51.88 ± 4.95 kPa, in ventral IA: 69.25 ± 2.59 kPa and in CEP: 70.82 ± 7.02 kPa) (Table 1). *Acan* ^*iE5/iE5*^ animals at E18.5 showed significant stiffening of the dorsal IA by E1: 1.97-fold and E2: 2.65-fold, the ventral IA by E1: 1.86-fold and E2: 1.99-fold, and the CEP by E1: 1.79 fold and E2: 1.97-fold compared to control (Table 1).

In general, we observed an embryonic stage-dependent increase of stiffness in all IVD compartments, independent of genotype, which correlates with the skeletal development and most likely caused by the maturation of collagen fibrils. Dorsal IA, ventral IA and CEP followed a gradual increase in Young’s moduli, reflecting their function in the IVD tissue. Interestingly, aggrecan-deficient mice lost the softer proteoglycan-related peak, and displayed significantly broader and stiffer Young’s moduli distribution than the corresponding control.

## 4 Discussion

The development of the axial skeleton is a complex process governed by intricate cellular signaling, resulting in the gradual differentiation of the vertebrae and the intervertebral discs. In this process, the extracellular matrix plays a critical role by providing the biochemical and biomechanical cues that guide the correct formation of specific skeletal elements. Aggrecan, the major proteoglycan of the cartilaginous ECM, is pivotal in determining the viscoelastic properties of the tissue and in dissipating mechanical stress. The IVD is a composite tissue whose compartments are composed of various combinations of ECM molecules, including collagens, proteoglycans and adhesive glycoproteins. Aggrecan is present in the nucleus pulposus, inner annulus fibrosus and cartilaginous endplate, where it is largely co-localized with type II collagen (Aszódi et al., 1998; Hayes, 2001; Shah et al., 2017). The clinical significance of aggrecan in the structure and function of the appendicular and axial skeleton is evidenced by the multitude of mutations and corresponding phenotypes that have been discovered in human and other species. The human and animal aggrecanopathies caused by mutations in the gene encoding ACAN are manifested in a wide spectrum of lethal and non-lethal skeletal chondrodysplasias (Gibson and Briggs, 2016). Spontaneously homozygous null mutations in chicken, mouse and cattle cause perinatal lethality with severe dwarfism and respiratory distress, indicating an essential function for ACAN in skeletal development (Li et al., 1993; Watanabe et al., 1994; Cavanagh et al., 2007). Neomorphic and hypomorphic mutations in *ACAN* are associated with short stature, early onset of osteoarthritis and multiple spinal abnormalities. IVD degeneration, the major cause of back pain, is associated with variable number of tandem repeats (VNTR) polymorphisms in exon 12 of *ACAN* coding for the CS1 gene domain (Mayer et al., 2013; Martirosyan et al., 2016). In the present study, we investigated the consequences of aggrecan depletion on the development of the IVDs in a novel mouse genetic model. *Acan* ^*iE5/iE5*^ mice, homozygous for an insertion in exon 5, develop perilethal dwarfism associated with severe abnormalities of the vertebral column. Mutants display premature ossification of the cervical vertebrae, deformed nucleus pulposus and disorganized inner annulus fibrosus and cartilaginous endplate. AFM imaging and indentation revealed abnormal collagen fibrils and increased stiffness in the forming CEP and IA.

By targeting the *Acan* locus in mouse embryonic stem cells, we introduced a single *loxP* site into exon 5, which encodes the hyaluronan-binding B segment of the globular, N-terminal G1 domain. The insertion disrupts the open reading frame, resulting in a premature stop codon in exon 5 and a hypothetic truncated protein of 263 amino acids. *Acan* ^*iE5/iE5*^ mice die at birth and are characterized by disproportionate dwarfism, cleft palate, protruding tongue and short snout, which is closely resembling to the phenotype of mice with cartilage matrix deficiency (*cmd*) (Rittenhouse et al., 1978). The *cmd* mice carry a 7 base pair deletion in exon 5 of *Acan* causing early termination of the translation in exon 6 and the formation of a 32 kDa truncated protein (Watanabe et al., 1994). Since both hypothetical truncated protein lack the functionally important GAG-carrying CS domains and the G3 domain important for multiple molecular interactions, we conclude that both *Acan* ^*iE5/iE5*^ and *Acan* ^*cmd/cmd*^ mice represent a functional null mutation of the murine *Acan* gene. Furthermore, the skeletal phenotype of the *Acan* ^*iE5/iE5*^ mice is very similar to that of another recessive, naturally occurring mouse model, *Acan* ^*cmd-Bc/cmd-Bc*^, which has a deletion of exons 2-18, the entire protein-coding sequence of the *Acan* gene (Krueger et al., 1999).

The null mutation in *Acan* ^*iE5/iE5*^ mice was confirmed at protein level. Immunoblotting of E18.5 knee cartilage extracts using antibody against the full length of rat ACAN demonstrated the absence of protein in homozygous mutant. Consequently, a significant reduction of 86.35% in sGAG content was recorded in the knee cartilage of *Acan* ^*iE5/iE5*^ mice, further confirming the functional significance of the insertional mutation. Furthermore, Safranin O staining for cartilage proteoglycans and GAGs demonstrated an intensive signal in cartilaginous tissues of the developing spine of wild type animals, but little if any staining was detected in the forming vertebral bodies and IVDs of *Acan* ^*iE5/iE5*^ mice. The residual sGAG observed in mutants may be due to the expression of other proteoglycans in cartilage. Versican is a large chondroitin sulfate proteoglycan of various extracellular matrices, which, similar to aggrecan, forms aggregates with hyaluronan (Nandadasa et al., 2014; Aspberg, 2016; Watanabe, 2022). During limb development, versican is strongly expressed during prechondrogenic mesenchymal condensation, downregulated in differentiated cartilage but its expression still present at the perichondrium and the articular cartilage surface. In rat, versican is immunolocalized to the cartilage of embryonic vertebral bodies and the inner annulus, while its expression is diminished in cartilaginous tissues at the neonate stage (Hayes, 2001). In addition to aggregating proteoglycans, members of the small leucine-rich repeat proteoglycans carrying dermatan (decorin, biglycan) or keratan sulfate (fibromodulin, lumican) GAG chains are also expressed in cartilaginous tissues (Roughley, 2006; Aspberg, 2016). It is important to note that using a G1 domain-specific antibody and immunohistochemistry, we observed a very faint staining of ACAN on sections through the developing cervical spine of *Acan* ^*iE5/iE5*^ mice. Since the insertional *iE5* targeting strategy may result in a hypothetical truncated protein representing the N-terminal part of the normal ACAN, we cannot exclude that our antibody detects the non-functional G1 globular domain of the aggrecan core protein.

Micro-CT analysis of *Acan* ^*iE5/iE5*^ mice at E18.5 revealed that aggrecan loss profoundly affects the morphogenesis of both the appendicular and the axial skeleton. In this study, we have focused on the development of the vertebral column and the intervertebral discs, which have been poorly characterized in the *Acan* ^*cmd/cmd*^ and *Acan* ^*cmd-Bc/cmd-Bc*^ mice. In accordance with previous murine immunolocalization studies (Aszódi et al., 1998; Hayes, 2001), aggrecan in wild type mice was strongly expressed at E12.5 in the NC sheath and moderately in the NC and PVs. At later embryonic stages, aggrecan was found in the cartilaginous VB, the CEP, NP and the IA indicating its important role in spine development. µCT scans and von Kossa staining in E18.5 mutant mice showed a compressed shape of the lumbar vertebrae and the premature ossification of the cervical vertebral bodies. Similar accelerated ossification of cervical vertebrae was reported in *Acan* ^*cmd-Bc/cmd-Bc*^ mice (Lauing et al., 2014), whereas joined calcification regions of the vertebral bodies and arches of the thoracic, lumbar and sacral vertebrae were described in *Acan* ^*cmd/cmd*^ mice (Rittenhouse et al., 1978). The premature ossification phenotype in mice may parallel some of the identified *ACAN* mutations in human, which are characterized by the manifestation of advanced bone age in prepubertal stages (Nilsson et al., 2014; Dateki et al., 2017; Gkourogianni et al., 2017). In nanomelic chick and *cmd-Bc* mice growth plates, altered pattern of typical endochondral differentiation molecules, (i.e., type X collagen, indian hedgehog, fibroblast growth factor and bone morphogenetic proteins) was found. The disruption of proper signaling may be the driving mechanism for early hypertrophic maturation of chondrocytes leading to long bone growth disorders. These findings suggest that an aggrecan-containing milieu is required for an appropriate morphogen gradient and consequent cell-ECM crosstalk and chondrocyte differentiation (Domowicz et al., 2009; Lauing et al., 2014).

Heterozygous *cmd* mice develop age-associated spine misalignment and IVD degeneration (Watanabe et al., 1997). *Acan* ^*cmd-Bc/cmd-Bc*^ (Lauing et al., 2014) and *Acan* ^*iE5/iE5*^ mice show cervical spine misalignment and abnormal positioning of the atlas and axis at E18.5. In our study, histological analysis of the cervical spine development revealed multiple abnormalities in the formation of the IVD tissues. During mouse development, the notochord contributes to the formation of the nucleus pulposus in between E12.5 and E18.5. Notochordal cells gradually vanish from the vertebral segments of the NC and expand at the intervertebral segments, forming a disc-like NP (Aszódi et al., 1998). In *Acan* ^*iE5/iE5*^ mice, this morphogenetic process is disturbed characterized by delayed removal of the notochordal cells from the vertebral segments between E14.5 and E18.5, and the rounded morphology of the NP at E18.5. Severely abnormal notochordal reorganization has been reported in mice with targeted mutation in *Col2a1* encoding type II collagen. *Col2a1* knockout mice die at birth and have a road-like notochord running through the vertebral column at E18.5, with notochordal cells unable to disappear at the level of vertebrae and to expand in between the vertebrae to form the NP (Aszódi et al., 1998). Furthermore, disorganization of the normal structure of the CEP and the IA are also common in *Col2a1* ^*−/−*^ and *Acan* ^*iE5/iE5*^ mice, implying that the two major macromolecular components of the cartilage ECM, aggrecan and type II collagen, are pivotal for the proper morphogenesis of the cartilaginous IVD tissues. Interestingly, heterozygous *Col2a1* ^*+/−*^ mice have a postnatal spine phenotype characterized by premature ossification of the CEP and decreased GAG content in the IVDs (Sahlman et al., 2001). In mice lacking type IX collagen, an important component of the heterotypic collagen fibrils in cartilage, disorganized CEP and age-associated IVD degeneration were observed (Kamper et al., 2016). Thus, these genetic studies in mice suggest that aggrecan and collagens play important role in IVD development during embryogenesis and in disc pathology in aging animals.

The pattern of type II collagen immunolocalization in *Acan* ^*iE5/iE5*^ mice were comparable to that of wild type mice, showing COL2A1 deposition in the NC sheath and PVs at E12.5, and in NC sheath, CEP, IA and VB at later stages. Type I collagen deposition was also similar between the genotypes with prominent expression in notochordal sheath at E12.5 and in the OA at E14.5-E18.5. Previous RT-PCR and *in situ* hybridization studies reported that *Col2a1* and *Col1a1* RNAs expression and distribution are also comparable in *cmd/cmd*, *cmd* ^*bc*^ */cmd* ^*bc*^ and wild type mice (Watanabe et al., 1997; Wai et al., 1998; Lauing et al., 2014). Similarly, the deposition of *Acan* did not change in the cartilaginous tissues of the vertebral column in *Col2a1* ^*−/−*^ mice (Aszódi et al., 1998). Interestingly, vertebral chondrocytes in *Col2a1* ^*−/−*^ mice ectopically express *Col1a1*, while we observed a very moderate and comparable deposition of type I collagen in the IA of wild type and *Acan* ^*iE5/iE5*^ mice only at E14.5. These cartilages thus represent an abnormal ECM composition, which either completely lacks aggrecan or type II collagen, with different outcome on the morphology, structure and biomechanical properties of the cartilaginous tissues. In *Acan* ^*iE5/iE5*^, *Acan* ^*cmd/cmd*^ and *Acan* ^*cmd-Bc/cmd-Bc*^ mice, the absence of aggrecan leads to shrinking of the cartilaginous tissues accompanied by increased cellular density and little, collapsed ECM between chondrocytes of the tracheal, limb and vertebral cartilages (Rittenhouse et al., 1978; Lauing et al., 2014). Importantly, quantification at E18.5 did not reveal difference in cell density in the NP and the ventral IA between wild type and *Acan* ^*iE5/iE5*^ mice, likely due to the moderate expression of aggrecan in these tissues compared to vertebral cartilage. In contrast, in *Col2a1* ^*−/−*^ mice, the cartilaginous VBs were enlarged, cell density was reduced, and the intervening ECM was increased (Aszódi et al., 1998). The opposing appearance of ACAN- and COL2A1-deficient cartilages is in agreement with the main properties of aggrecan and type II collagen in the tissue. ACAN depletion largely removes the cushioning proteoglycans between collagen fibrils, resulting in collapse of the collagenous ECM and a decrease of tissue volume, whereas the absence of collagen fibrils allows for the expansion of the highly hydrated proteoglycan moiety and an increase in tissue volume.

Analysis of high-resolution AFM images demonstrated an apparently denser network of collagen fibrils in the cartilaginous CEP and IA of *Acan* ^*iE5/iE5*^ mice compared to wild type. Consistent decrease in the diameter of collagen fibrils was also found in all cartilaginous IVD tissues of *Acan* ^*iE5/iE5*^ mutants. This finding is in contrast to previous observations obtained by electron microscopy in *cmd/cmd* mice, which reported thicker collagen fibrils in tracheal or epiphyseal cartilage compared with wild type (Rittenhouse et al., 1978; Kobayakawa et al., 1985). The reason of this discrepancy is not known. On one hand, we cannot exclude tissue specific differences in collagen fibril maturation between the developing IVD tissues and tracheal/epiphyseal cartilages in *Acan* ^*iE5/iE5*^ mice. On the other hand, transmission electron microscopy may introduce artefacts to the collagenous network, which may differ according to the composition of the examined ECM, due to the harsh chemical fixation and tissue processing of the cartilage samples (Hunziker et al., 2014). In the present study, we obtained high-resolution AFM images on native tissue cryo-sections, which may better preserve and visualize the collagen network (Graham et al., 2010), especially the population of nanofibrils, which usually do not survive chemical fixation (Hunziker et al., 2014).

The role of aggregating (aggrecan and versican) and non-aggregating proteoglycans in modulation of collagen fibrillogenesis is controversial. The thick fibrils observed in epiphyseal cartilage of *cmd/cmd* mice by electron microscopy (Rittenhouse et al., 1978; Kobayakawa et al., 1985) and the thinner collagen fibrils found in the cartilaginous IA and CEP of *Acan* ^*iE5/iE5*^ mice by AFM suggest that aggrecan either inhibits or accelerates lateral growth of collagen fibrils. It has been previously shown that both aggregating and non-aggregating proteoglycans isolated from young human cartilage (0–3 months) increase fibrillogenesis of type II collagen *in vitro*, which was less effective after the removal of CS chains with chondroitinase ABC. On the other hand, only fraction of aggregating proteoglycans isolated from 18 years-old knee cartilage enhanced type II collagen fibrillogenesis, which was not affected by chondroitinase ABC treatment (Kuijer et al., 1988). Heparin sulfate/chondroitin sulfate (HS/CS)-substituted perlecan is a non-aggregating proteoglycan present in embryonic growth plate cartilages. Recombinant HS/CS perlecan enhanced types I and II collagen fibrillogenesis on a CS-dependent manner (Kvist et al., 2006). A more recent study employing *in vitro* turbidity assay and scanning electron microscopy of type I collagen matrices with or without CS proteoglycans demonstrated that versican enhanced collagen fibrillogenesis and increased collagen fibril diameter, whereas aggrecan slightly slowed fibrillogenesis but had no effect on fiber diameter (Chen et al., 2020). The SLRPs decorin and lumican decreased the rate of collagen fibrillogenesis, and decorin decreased collagen fibril diameter (Chen et al., 2020) confirming previous *in vitro* study showing that SLRPs rather than aggrecan inhibit lateral aggregation of tendon extracted collagen fibrils (Vogel and Trotter, 1987). These later findings therefore suggest that aggrecan has no major direct modulatory role in fibrillogenesis and fibril diameter regulation. Rather, we hypothesize that in the absence of aggrecan *in vivo*, SLRPs and versican may have facilitated access to the forming collagen fibrils and, in turn, exert their role for the lateral growth of the fibrils. Quantitative analysis of the ratio of collagens and these proteoglycans in the cartilaginous IVD tissues of *Acan* ^*iE5/iE5*^ mice, could eventually help to identify the mechanism behind the abnormal fibril diameters.

The most interesting and novel phenotype observed in this study is that loss of aggrecan markedly alters the biomechanical properties of IVD tissues. IT-AFM is the state-of-the-art tool for characterizing ECM biomechanics. By using sharp, nanoscaled pyramidal probing tips, the method is able to resolve the stiffer collagenous and softer proteoglycan phases of cartilaginous tissues (Loparic et al., 2010; Prein et al., 2016). *Acan* ^*iE5/iE5*^ IVD tissues exhibited wider range of Young’s moduli with a marked shift towards stiffer values compared to wild type. Importantly, the typical proteoglycan peak (E1) recorded in control was right-shifted in all compartments of the developing IVD in mutant mice, indicating that aggrecan is the key component of the soft, gel-like intrafibrillar ECM that allows the tissue to resist compressive forces. These results are consistent with the shift towards stiffer Young’s moduli observed in the articular cartilage of hypomorphic *Agc1* ^*CreERT2/CreERT2*^ mice (Alberton et al., 2019). In aggrecan mutant mice, the changes towards a harder cartilaginous ECM are attributable to the disrupted balance between the swelling pressure exerted by proteoglycans and the tensile forces of the collagen network. With the loss of aggrecan, there is a tremendous decrease in sGAG with negative charge density, which consequently impairs tissue hydration and reduces swelling pressure, resulting in a collapsed and stiff collagen-dominated matrix. In line with our findings, interleukin-1α -induced aggrecanolysis in *ex vivo* mouse femoral head results in an increase in the nanostiffness of the superficial cartilage, indicating that aggrecan depletion has exposed more collagen fibrils available to the AFM tip, which is inherently stiffer (Uddin et al., 2017). Of note is that we observed a gradual increase in matrix stiffness in all IVD compartments with development in both wild type and *Acan* ^*iE5/iE5*^ IVD mice. We hypothesize that this developmental stage-specific stiffening of the ECM is due to the maturation and increased synthesis of collagen fibrils, as previously suggested for the developing growth plate cartilage (Prein et al., 2016).

We previously proposed that the lack of notochordal reorganization and IVD formation in *Col2a1* ^*−/−*^ mice is a consequence of the reduced biomechanical forces acting on the notochord, normally exerted by the developing vertebral bodies (Aszódi et al., 1998). In the absence of type II collagen, the cartilage has a gel-like appearance and is likely to have a softer ECM, which is insufficient to squeeze notochordal cells into the intervertebral segments to form the nucleus pulposus. Recently, we have also shown that IVD degeneration in aged collagen IX-deficient mice is associated with reduced nanostiffness of the cartilaginous endplate and inner annulus fibrosus in newborn (Kamper et al., 2016). In both collagen-deficient mouse models and the *Acan* ^*iE5/iE5*^ mice, alteration in ECM biomechanics is ultimately one of the possible causes leading to failure of the mechanically anisotropic vertebral column to form, compromising proper IVD development and function. In this respect, the failure in forming elliptical shaped NP in *Acan* ^*iE5/iE5*^ mice may be a consequence of an abnormally stiffer IA that counteracts the timely dislocation of notochordal cells from the vertebral segments and their anisotropic, lateral expansion between vertebrae. As an alternative to the “pressure model”, the movement of notochordal cells into the nucleus pulposus can be guided by the deposition of attractant/repulsive proteins (Lawson and Harfe, 2015). Since aggrecan plays a critical role in both determining the mechanical properties of the ECM and modulating the gradient of morphogens in cartilage tissues, this suggests that either of these mechanisms may be impaired in *Acan* ^*iE5/iE5*^ *mice*, leading to abnormal spine development.

## Data Availability

The original contributions presented in the study are included in the article/supplementary material, further inquiries can be directed to the corresponding author.
